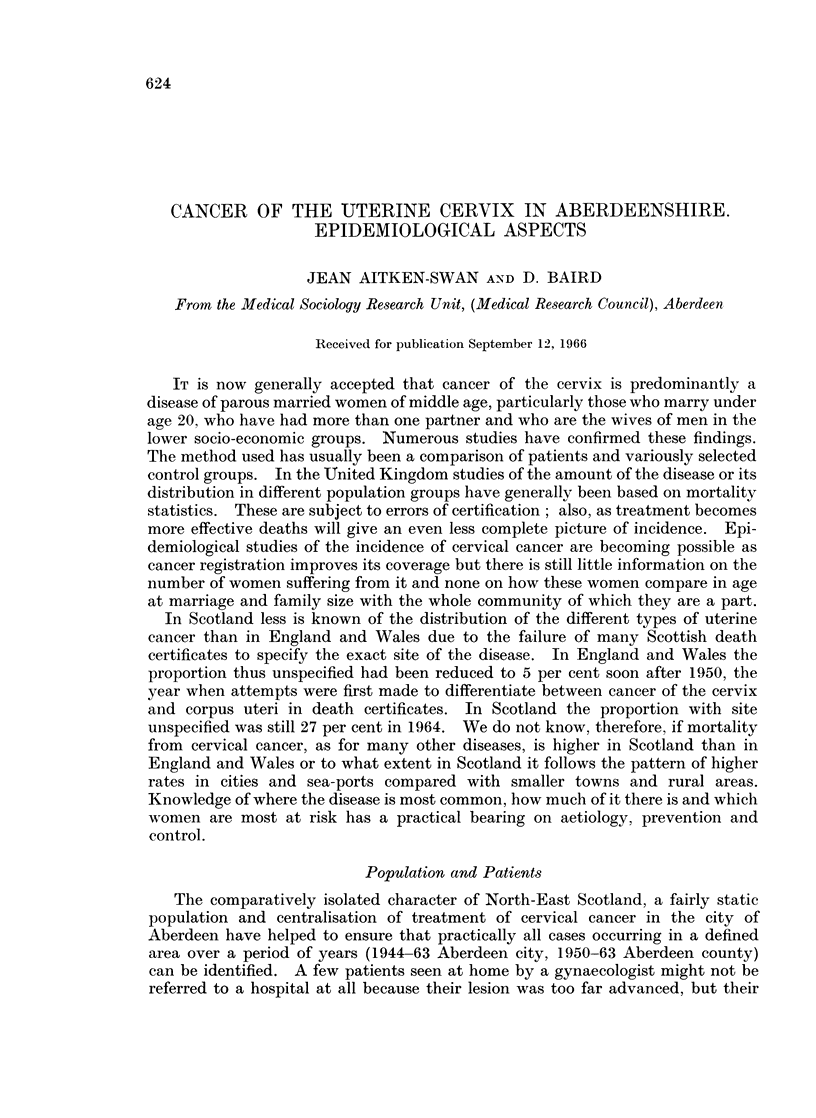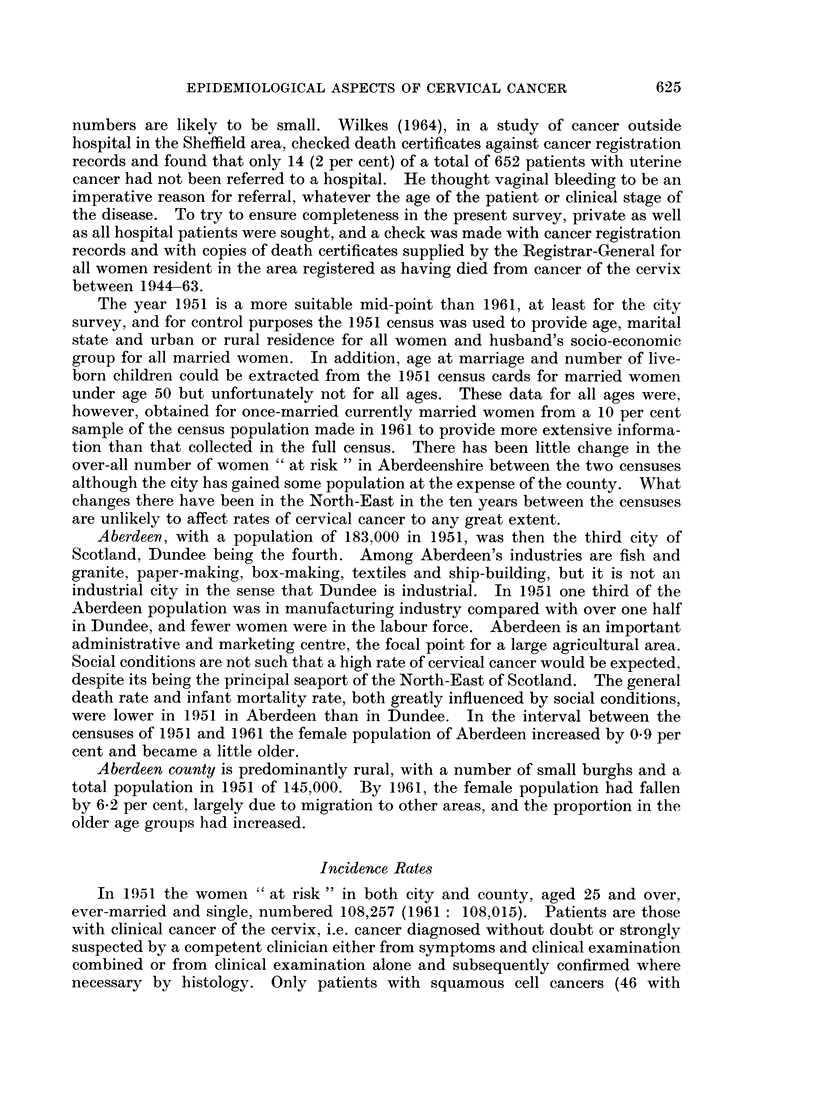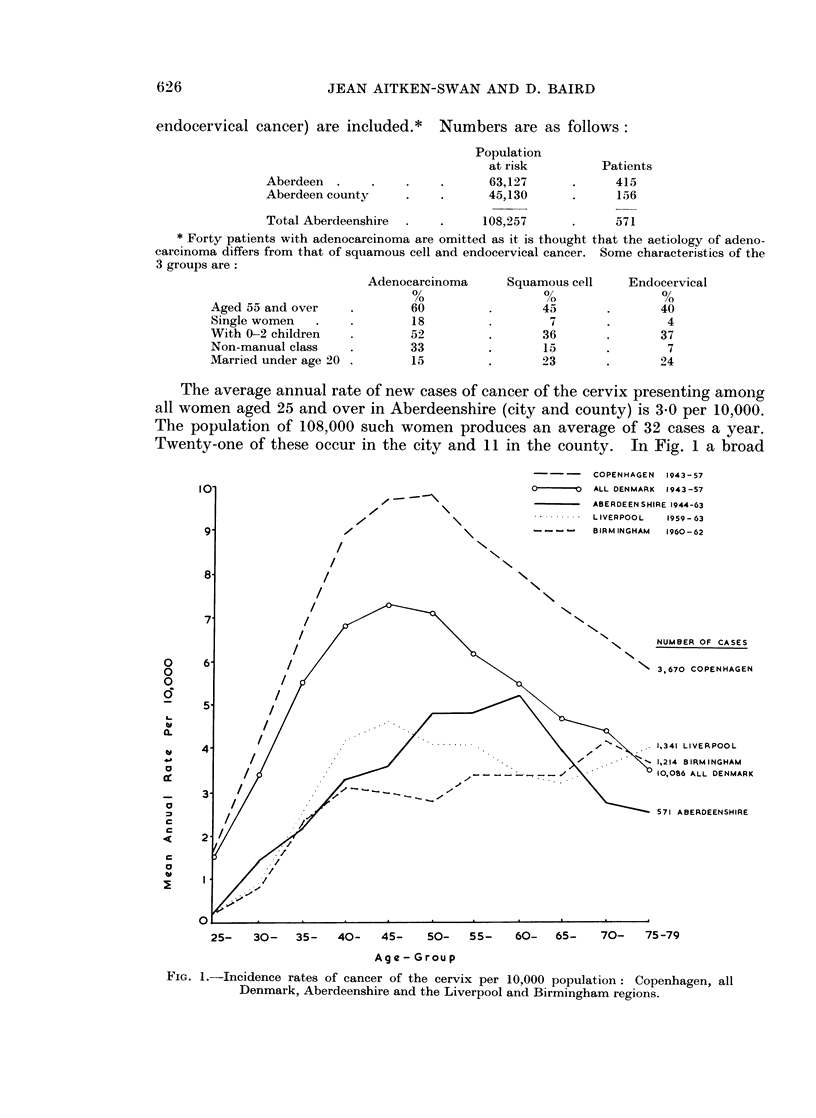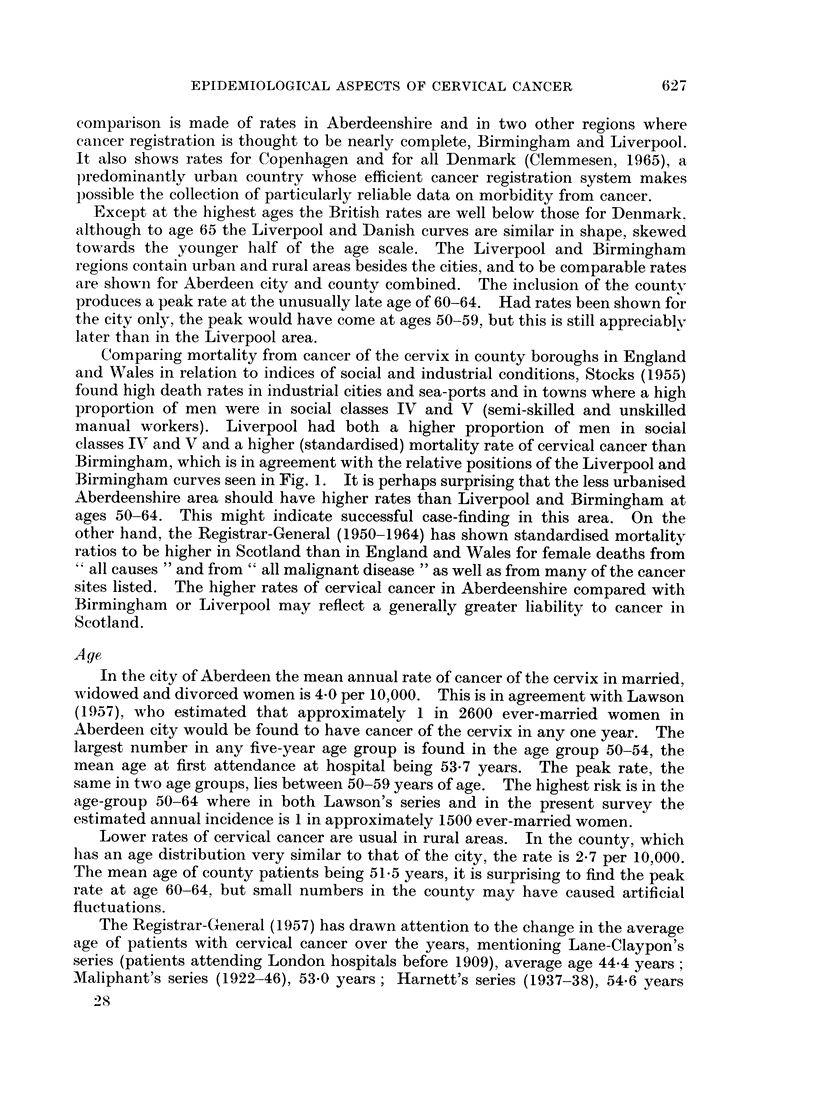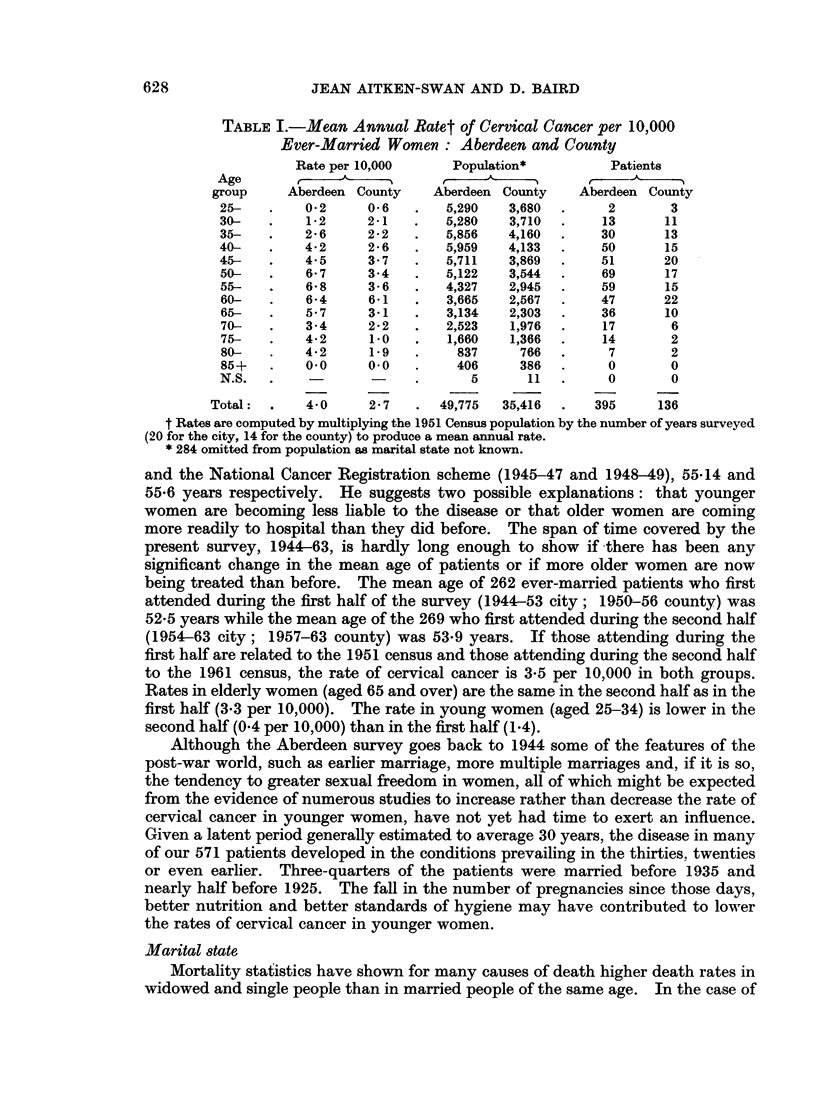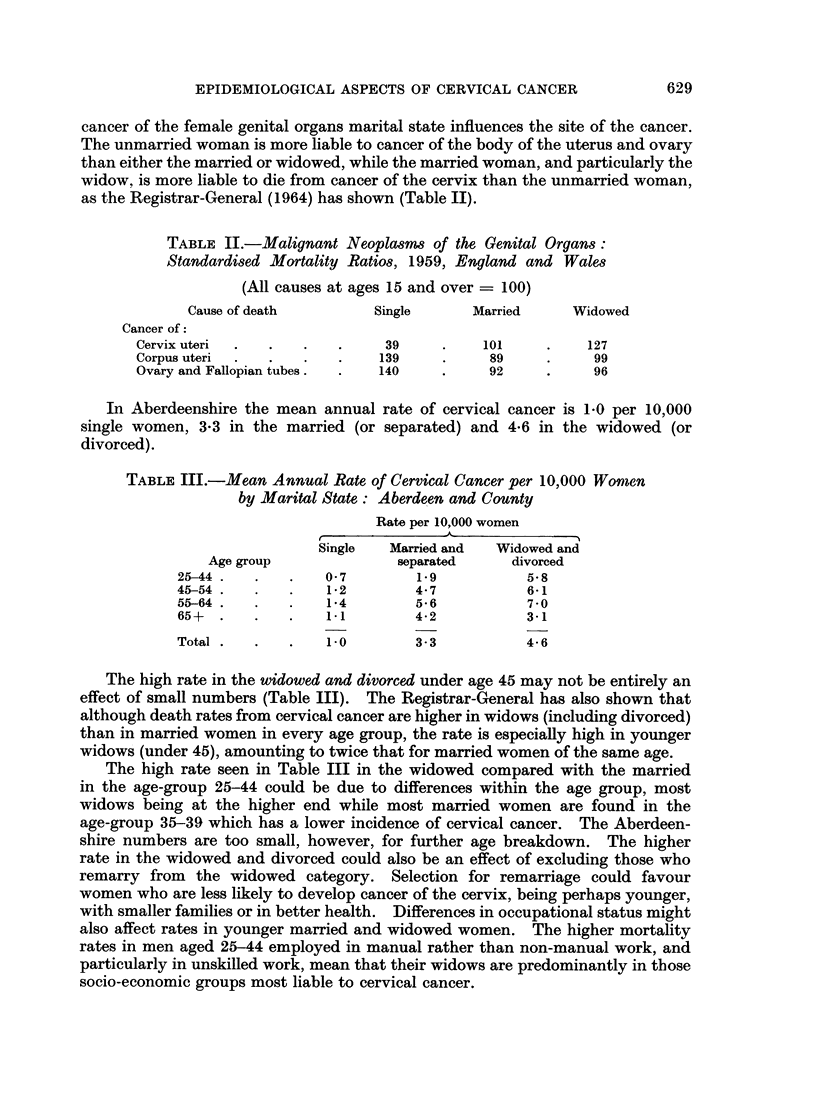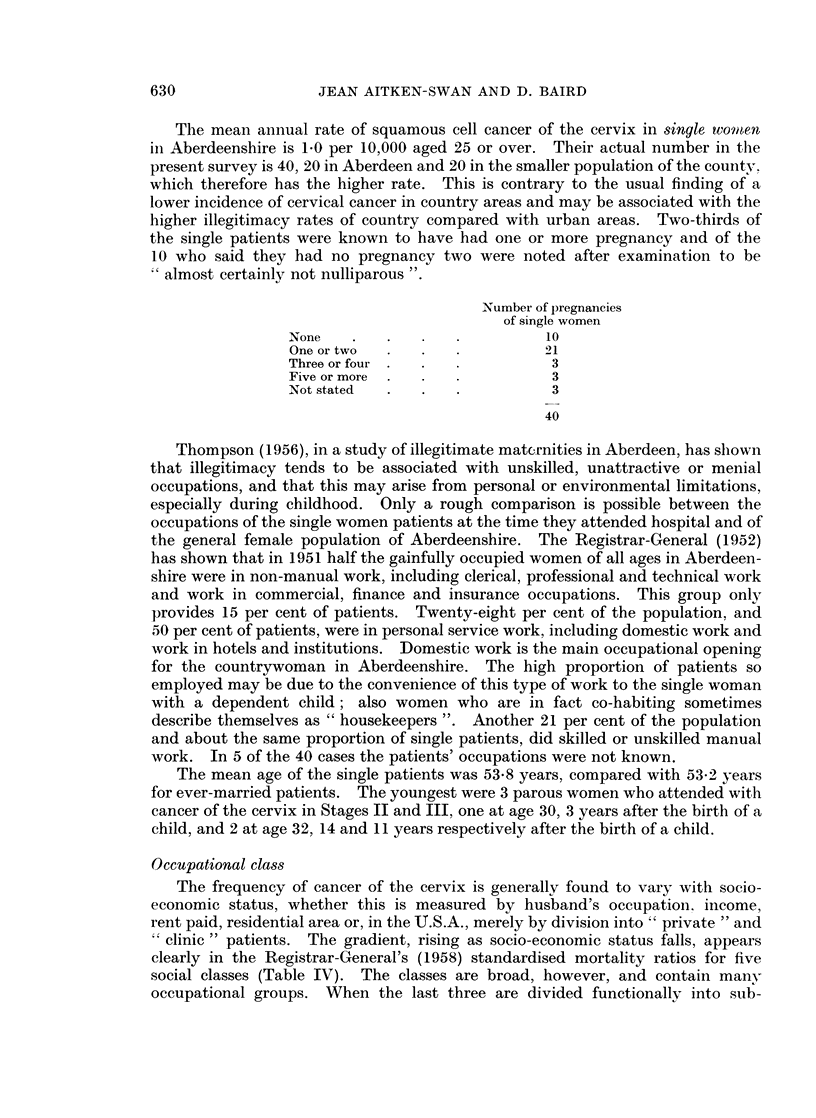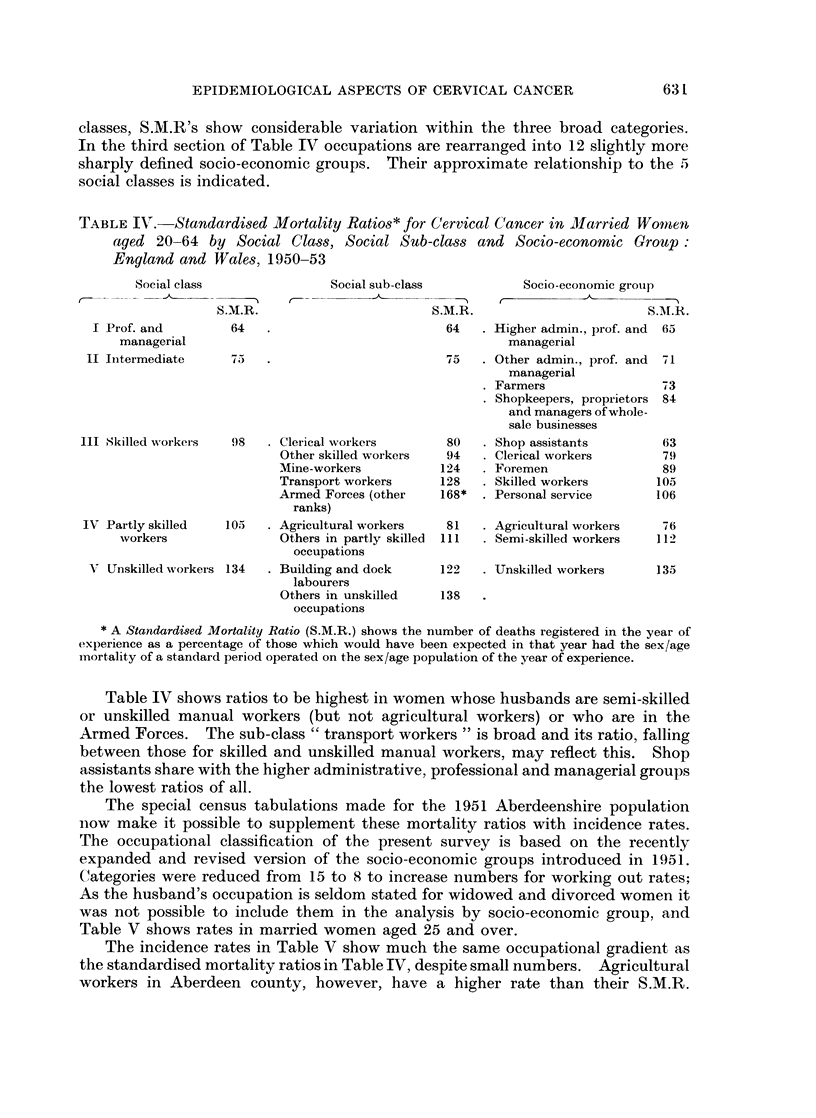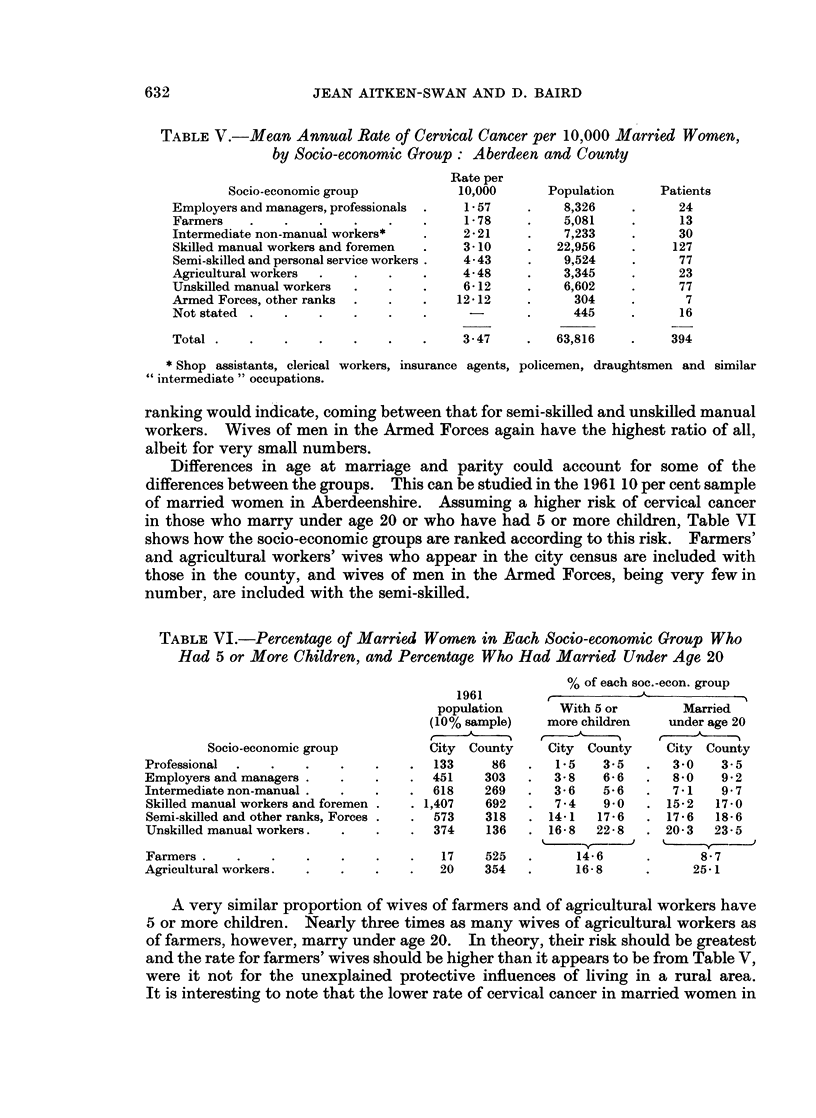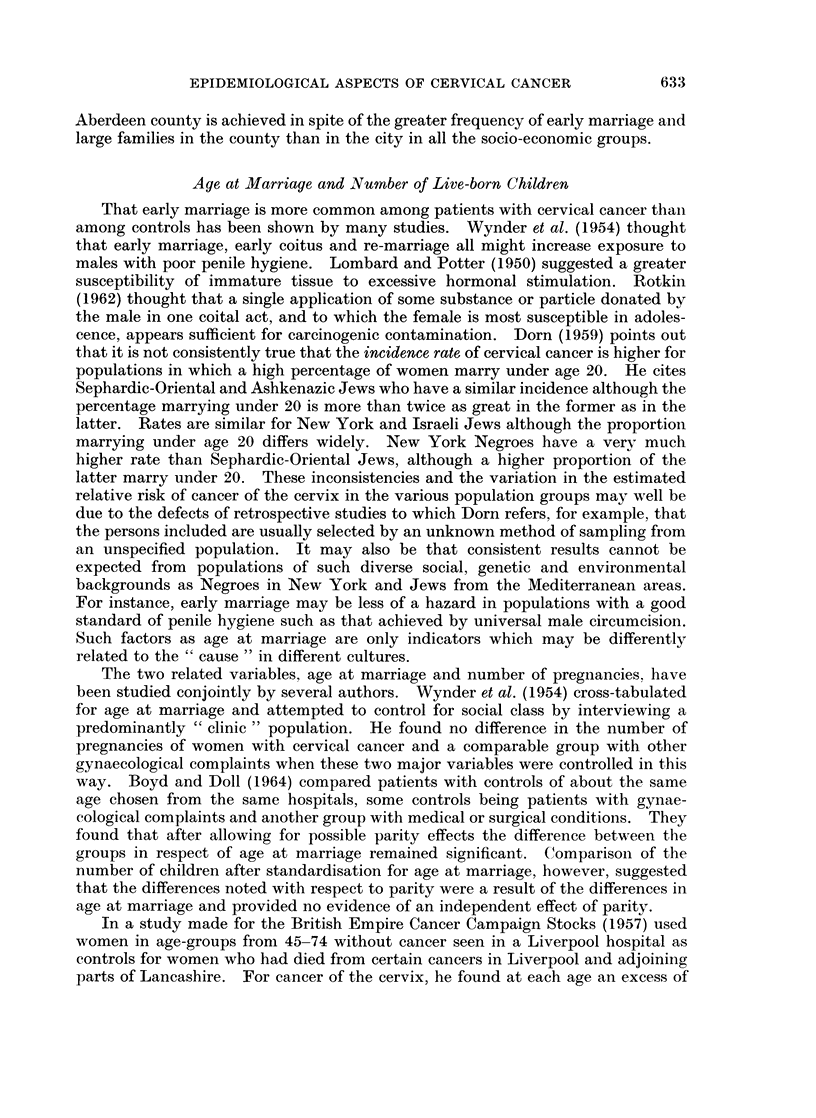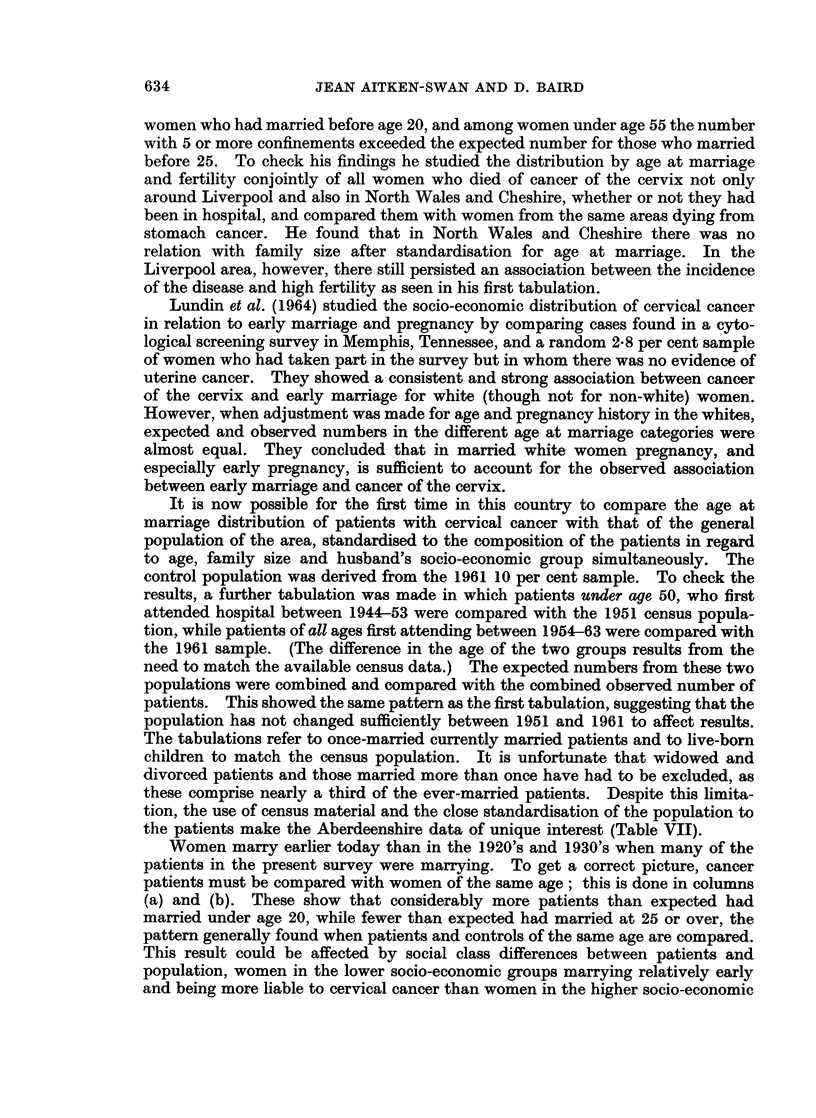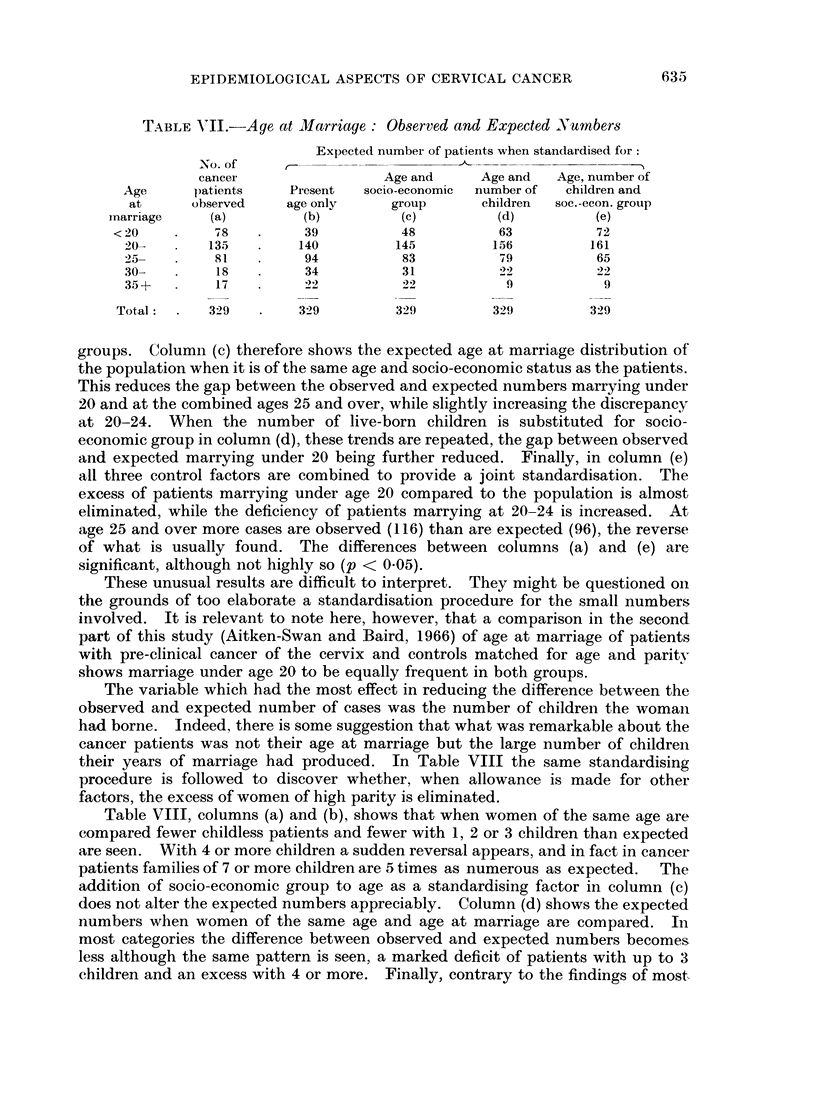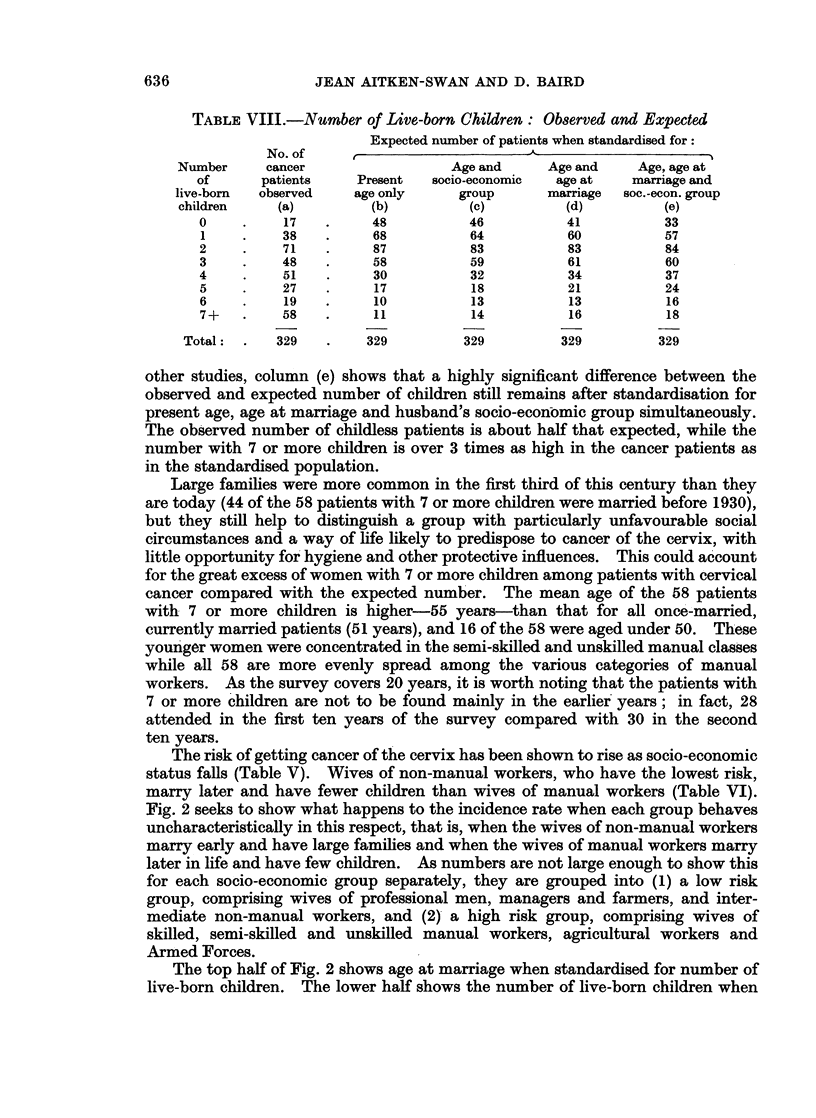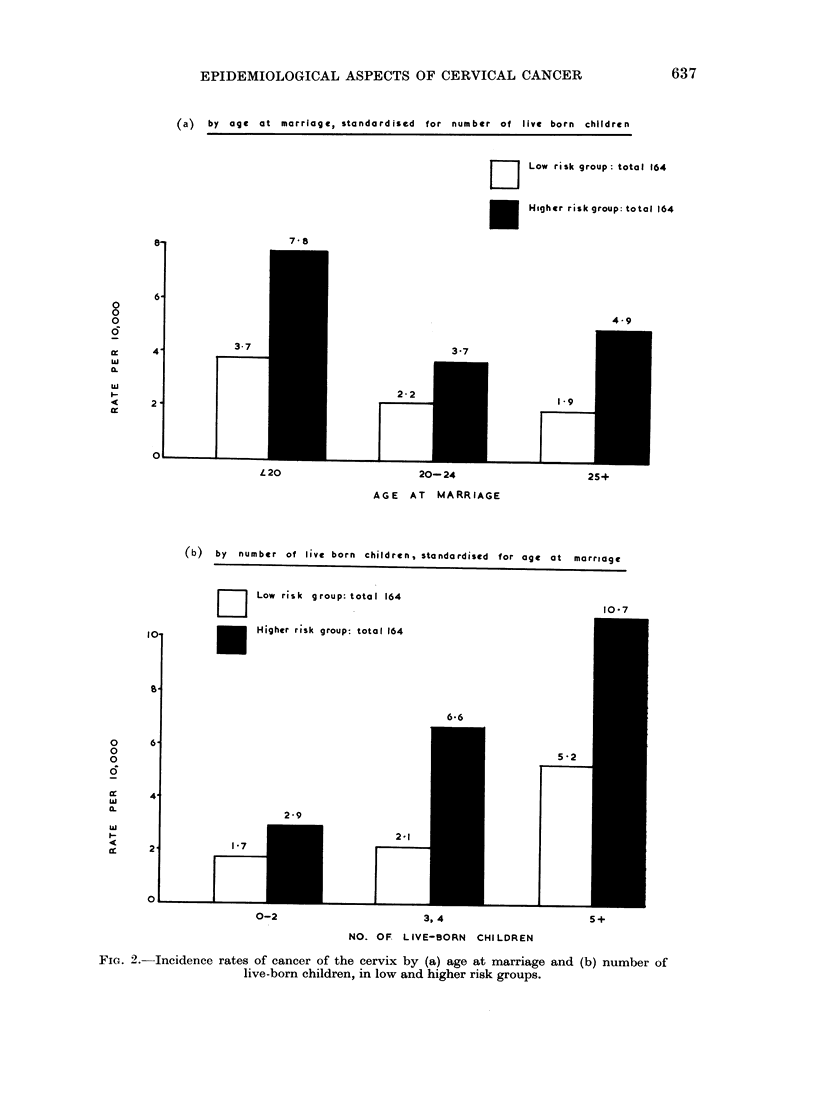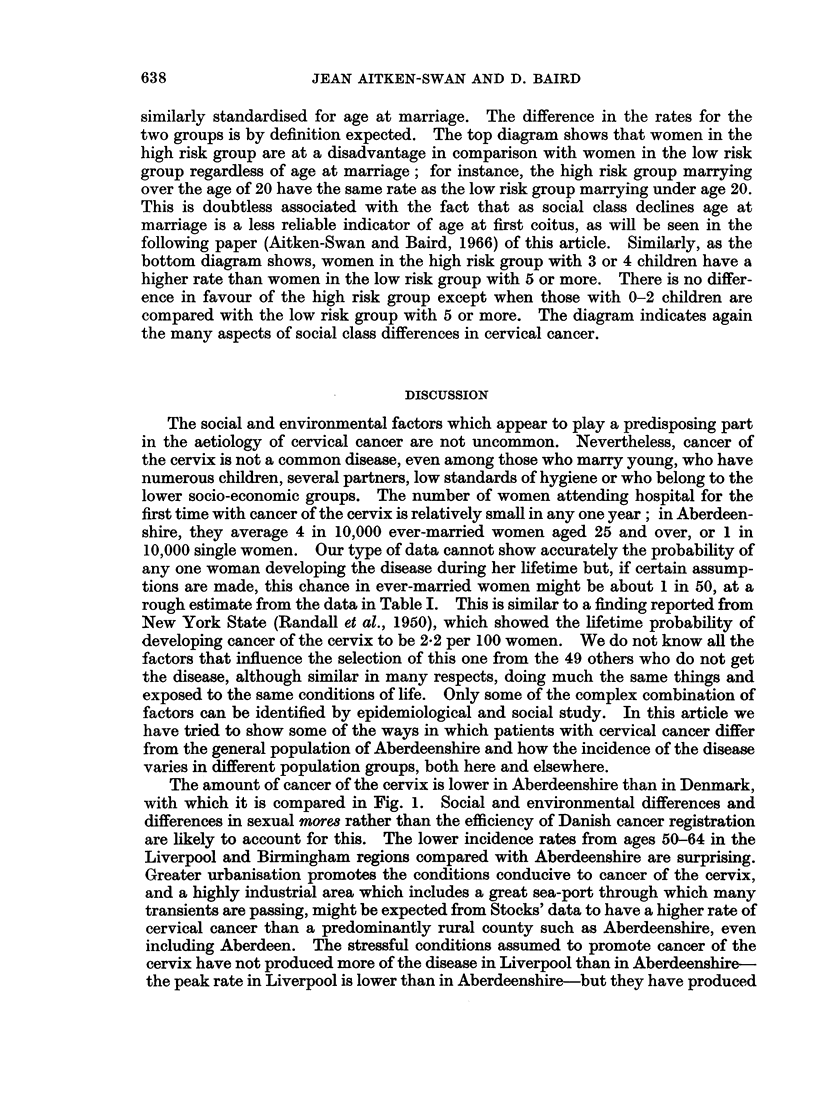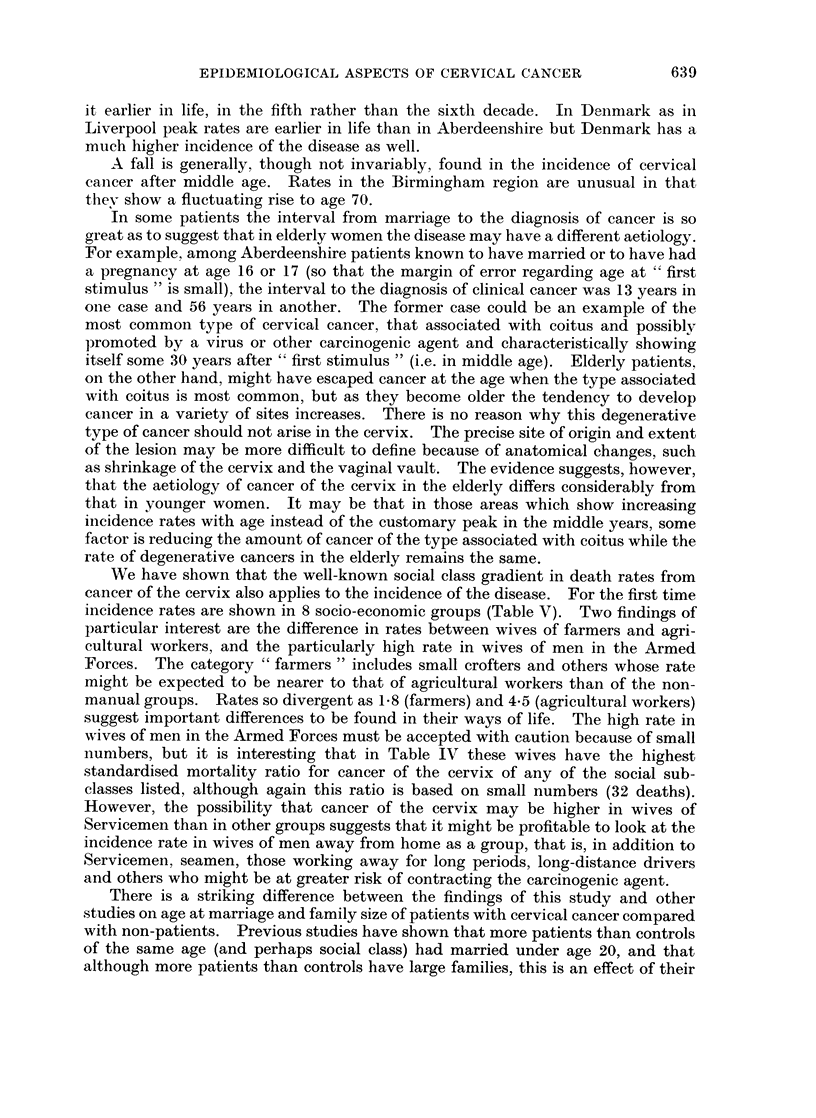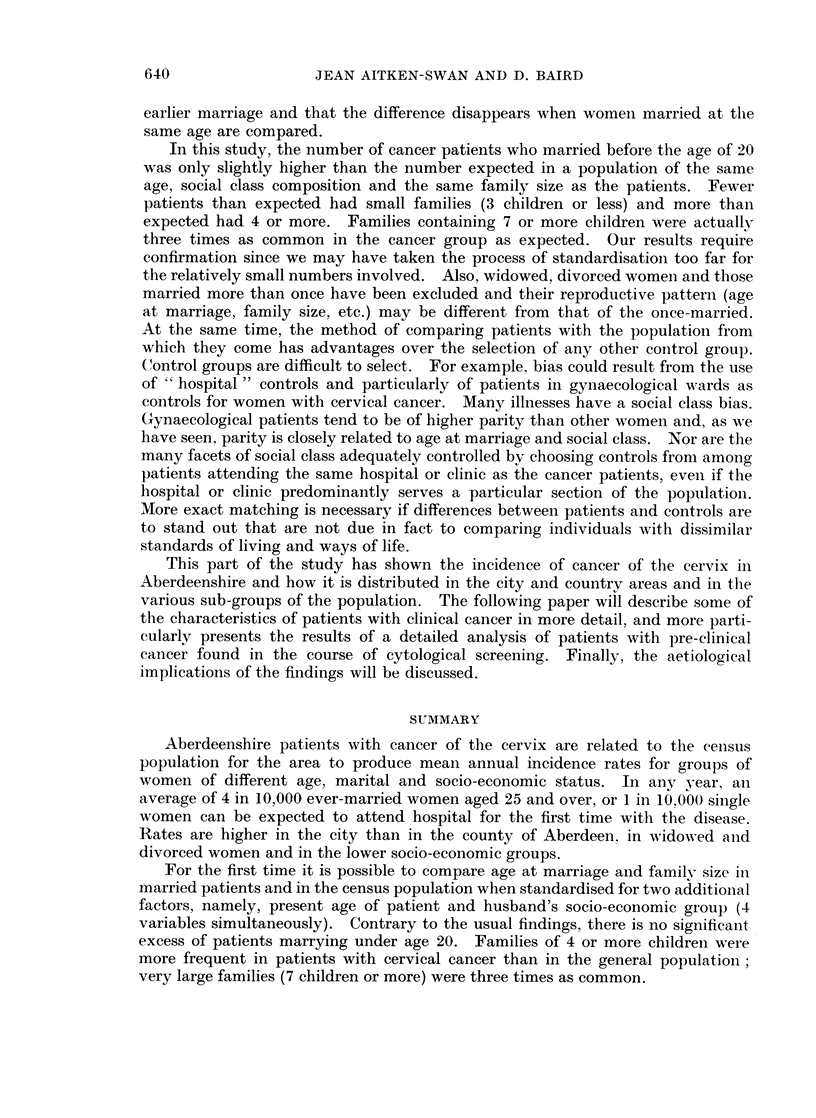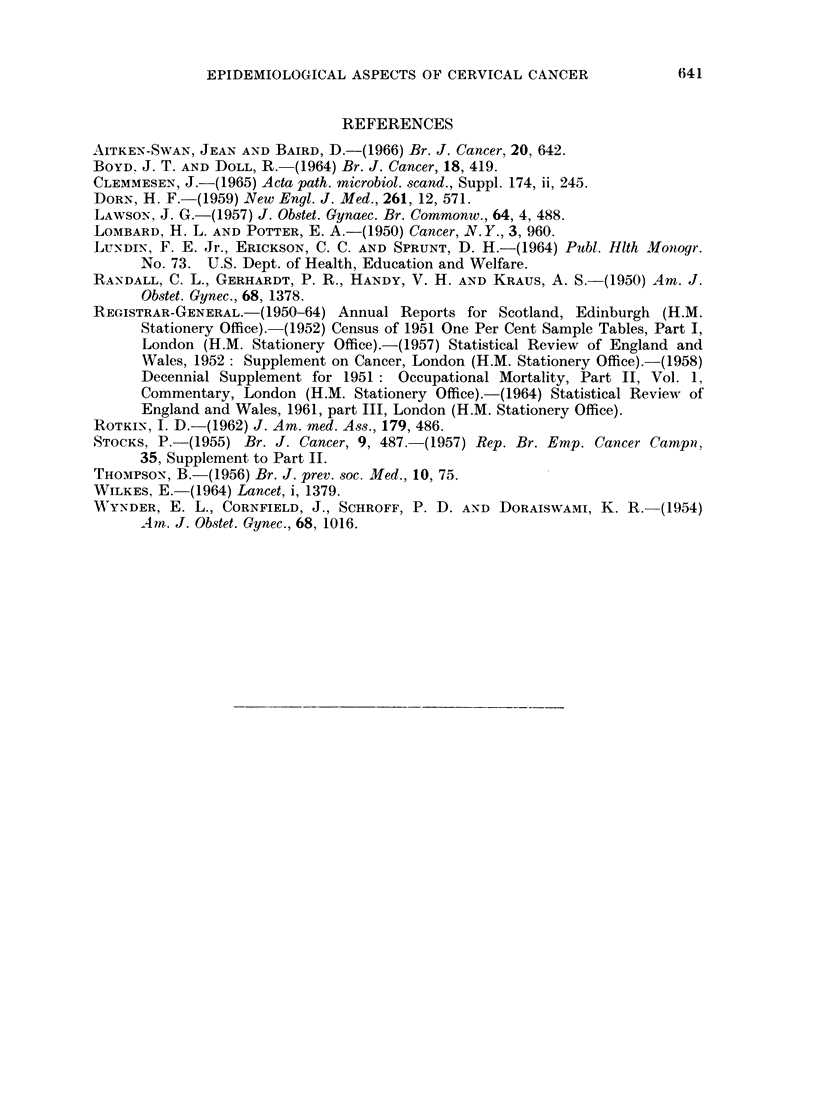# Cancer of the uterine cervix in Aberdeenshire. Epidemiological aspects.

**DOI:** 10.1038/bjc.1966.75

**Published:** 1966-12

**Authors:** J. Aitken-Swan, D. Baird


### CANCER OF THE UTERINE CERVIX IN ABERDEENSHIRE. EPIDEMIOLOGICAL ASPECTS

JEAN AITKEN-SWAN AND D. BAIRD

*From the Medical Sociology Research Unit, (Medical Research Council), Aberdeen*

Received for publication September 12, 1966

It is now generally accepted that cancer of the cervix is predominantly a disease of parous married women of middle age, particularly those who marry under age 20, who have had more than one partner and who are the wives of men in the lower socio-economic groups. Numerous studies have confirmed these findings. The method used has usually been a comparison of patients and variously selected control groups. In the United Kingdom studies of the amount of the disease or its distribution in different population groups have generally been based on mortality statistics. These are subject to errors of certification ; also, as treatment becomes more effective deaths will give an even less complete picture of incidence. Epidemiological studies of the incidence of cervical cancer are becoming possible as cancer registration improves its coverage but there is still little information on the number of women suffering from it and none on how these women compare in age at marriage and family size with the whole community of which they are a part.

In Scotland less is known of the distribution of the different types of uterine cancer than in England and Wales due to the failure of many Scottish death certificates to specify the exact site of the disease. In England and Wales the proportion thus unspecified had been reduced to 5 per cent soon after 1950, the year when attempts were first made to differentiate between cancer of the cervix and corpus uteri in death certificates. In Scotland the proportion with site unspecified was still 27 per cent in 1964. We do not know, therefore, if mortality from cervical cancer, as for many other diseases, is higher in Scotland than in England and Wales or to what extent in Scotland it follows the pattern of higher rates in cities and sea-ports compared with smaller towns and rural areas. Knowledge of where the disease is most common, how much of it there is and which women are most at risk has a practical bearing on aetiology, prevention and control.

### *Population and Patients*

The comparatively isolated character of North-East Scotland, a fairly static population and centralisation of treatment of cervical cancer in the city of Aberdeen have helped to ensure that practically all cases occurring in a defined area over a period of years (1944–63 Aberdeen city, 1950–63 Aberdeen county) can be identified. A few patients seen at home by a gynaecologist might not be referred to a hospital at all because their lesion was too far advanced, but their numbers are likely to be small. Wilkes (1964), in a study of cancer outside hospital in the Sheffield area, checked death certificates against cancer registration records and found that only 14 (2 per cent) of a total of 652 patients with uterine cancer had not been referred to a hospital. He thought vaginal bleeding to be an imperative reason for referral, whatever the age of the patient or clinical stage of the disease. To try to ensure completeness in the present survey, private as well as all hospital patients were sought, and a check was made with cancer registration records and with copies of death certificates supplied by the Registrar-General for all women resident in the area registered as having died from cancer of the cervix between 1944–63.

The year 1951 is a more suitable mid-point than 1961, at least for the city survey, and for control purposes the 1951 census was used to provide age, marital state and urban or rural residence for all women and husband's socio-economic group for all married women. In addition, age at marriage and number of live-born children could be extracted from the 1951 census cards for married women under age 50 but unfortunately not for all ages. These data for all ages were, however, obtained for once-married currently married women from a 10 per cent sample of the census population made in 1961 to provide more extensive information than that collected in the full census. There has been little change in the over-all number of women " at risk " in Aberdeenshire between the two censuses although the city has gained some population at the expense of the county. What changes there have been in the North-East in the ten years between the censuses are unlikely to affect rates of cervical cancer to any great extent.

*Aberdeen*, with a population of 183,000 in 1951, was then the third city of Scotland, Dundee being the fourth. Among Aberdeen's industries are fish and granite, paper-making, box-making, textiles and ship-building, but it is not an industrial city in the sense that Dundee is industrial. In 1951 one third of the Aberdeen population was in manufacturing industry compared with over one half in Dundee, and fewer women were in the labour force. Aberdeen is an important administrative and marketing centre, the focal point for a large agricultural area. Social conditions are not such that a high rate of cervical cancer would be expected, despite its being the principal seaport of the North-East of Scotland. The general death rate and infant mortality rate, both greatly influenced by social conditions, were lower in 1951 in Aberdeen than in Dundee. In the interval between the censuses of 1951 and 1961 the female population of Aberdeen increased by 0·9 per cent and became a little older.

*Aberdeen county* is predominantly rural, with a number of small burghs and a total population in 1951 of 145,000. By 1961, the female population had fallen by 6·2 per cent, largely due to migration to other areas, and the proportion in the older age groups had increased.

### *Incidence Rates*

In 1951 the women " at risk " in both city and county, aged 25 and over, ever-married and single, numbered 108,257 (1961 : 108,015). Patients are those with clinical cancer of the cervix, i.e. cancer diagnosed without doubt or strongly suspected by a competent clinician either from symptoms and clinical examination combined or from clinical examination alone and subsequently confirmed where necessary by histology. Only patients with squamous cell cancers (46 with endocervical cancer) are included.* Numbers are as follows :

| | Population at risk | Patients |
|---|---|---|
| Aberdeen | 63,127 | 415 |
| Aberdeen county | 45,130 | 156 |
| Total Aberdeenshire | 108,257 | 571 |

* Forty patients with adenocarcinoma are omitted as it is thought that the aetiology of adenocarcinoma differs from that of squamous cell and endocervical cancer. Some characteristics of the 3 groups are :

| | Adenocarcinoma % | Squamous cell % | Endocervical % |
|---|---|---|---|
| Aged 55 and over | 60 | 45 | 40 |
| Single women | 18 | 7 | 4 |
| With 0–2 children | 52 | 36 | 37 |
| Non-manual class | 33 | 15 | 7 |
| Married under age 20 | 15 | 23 | 24 |

The average annual rate of new cases of cancer of the cervix presenting among all women aged 25 and over in Aberdeenshire (city and county) is 3·0 per 10,000. The population of 108,000 such women produces an average of 32 cases a year. Twenty-one of these occur in the city and 11 in the county. In Fig. 1 a broad

FIG. 1.—Incidence rates of cancer of the cervix per 10,000 population : Copenhagen, all Denmark, Aberdeenshire and the Liverpool and Birmingham regions.

comparison is made of rates in Aberdeenshire and in two other regions where cancer registration is thought to be nearly complete, Birmingham and Liverpool. It also shows rates for Copenhagen and for all Denmark (Clemmesen, 1965), a predominantly urban country whose efficient cancer registration system makes possible the collection of particularly reliable data on morbidity from cancer.

Except at the highest ages the British rates are well below those for Denmark, although to age 65 the Liverpool and Danish curves are similar in shape, skewed towards the younger half of the age scale. The Liverpool and Birmingham regions contain urban and rural areas besides the cities, and to be comparable rates are shown for Aberdeen city and county combined. The inclusion of the county produces a peak rate at the unusually late age of 60–64. Had rates been shown for the city only, the peak would have come at ages 50–59, but this is still appreciably later than in the Liverpool area.

Comparing mortality from cancer of the cervix in county boroughs in England and Wales in relation to indices of social and industrial conditions, Stocks (1955) found high death rates in industrial cities and sea-ports and in towns where a high proportion of men were in social classes IV and V (semi-skilled and unskilled manual workers). Liverpool had both a higher proportion of men in social classes IV and V and a higher (standardised) mortality rate of cervical cancer than Birmingham, which is in agreement with the relative positions of the Liverpool and Birmingham curves seen in Fig. 1. It is perhaps surprising that the less urbanised Aberdeenshire area should have higher rates than Liverpool and Birmingham at ages 50–64. This might indicate successful case-finding in this area. On the other hand, the Registrar-General (1950–1964) has shown standardised mortality ratios to be higher in Scotland than in England and Wales for female deaths from " all causes " and from " all malignant disease " as well as from many of the cancer sites listed. The higher rates of cervical cancer in Aberdeenshire compared with Birmingham or Liverpool may reflect a generally greater liability to cancer in Scotland.

*Age*

In the city of Aberdeen the mean annual rate of cancer of the cervix in married, widowed and divorced women is 4·0 per 10,000. This is in agreement with Lawson (1957), who estimated that approximately 1 in 2600 ever-married women in Aberdeen city would be found to have cancer of the cervix in any one year. The largest number in any five-year age group is found in the age group 50–54, the mean age at first attendance at hospital being 53·7 years. The peak rate, the same in two age groups, lies between 50–59 years of age. The highest risk is in the age-group 50–64 where in both Lawson's series and in the present survey the estimated annual incidence is 1 in approximately 1500 ever-married women.

Lower rates of cervical cancer are usual in rural areas. In the county, which has an age distribution very similar to that of the city, the rate is 2·7 per 10,000. The mean age of county patients being 51·5 years, it is surprising to find the peak rate at age 60–64, but small numbers in the county may have caused artificial fluctuations.

The Registrar-General (1957) has drawn attention to the change in the average age of patients with cervical cancer over the years, mentioning Lane-Claypon's series (patients attending London hospitals before 1909), average age 44·4 years ; Maliphant's series (1922–46), 53·0 years ; Harnett's series (1937–38), 54·6 years

TABLE I.—*Mean Annual Rate*† *of Cervical Cancer per* 10,000 *Ever-Married Women : Aberdeen and County*

| Age group | Rate per 10,000 Aberdeen | Rate per 10,000 County | Population* Aberdeen | Population* County | Patients Aberdeen | Patients County |
|---|---|---|---|---|---|---|
| 25– | 0·2 | 0·6 | 5,290 | 3,680 | 2 | 3 |
| 30– | 1·2 | 2·1 | 5,280 | 3,710 | 13 | 11 |
| 35– | 2·6 | 2·2 | 5,856 | 4,160 | 30 | 13 |
| 40– | 4·2 | 2·6 | 5,959 | 4,133 | 50 | 15 |
| 45– | 4·5 | 3·7 | 5,711 | 3,869 | 51 | 20 |
| 50– | 6·7 | 3·4 | 5,122 | 3,544 | 69 | 17 |
| 55– | 6·8 | 3·6 | 4,327 | 2,945 | 59 | 15 |
| 60– | 6·4 | 6·1 | 3,665 | 2,567 | 47 | 22 |
| 65– | 5·7 | 3·1 | 3,134 | 2,303 | 36 | 10 |
| 70– | 3·4 | 2·2 | 2,523 | 1,976 | 17 | 6 |
| 75– | 4·2 | 1·0 | 1,660 | 1,366 | 14 | 2 |
| 80– | 4·2 | 1·9 | 837 | 766 | 7 | 2 |
| 85+ | 0·0 | 0·0 | 406 | 386 | 0 | 0 |
| N.S. | — | — | 5 | 11 | 0 | 0 |
| Total: | 4·0 | 2·7 | 49,775 | 35,416 | 395 | 136 |

† Rates are computed by multiplying the 1951 Census population by the number of years surveyed (20 for the city, 14 for the county) to produce a mean annual rate.

* 284 omitted from population as marital state not known.

and the National Cancer Registration scheme (1945–47 and 1948–49), 55·14 and 55·6 years respectively. He suggests two possible explanations : that younger women are becoming less liable to the disease or that older women are coming more readily to hospital than they did before. The span of time covered by the present survey, 1944–63, is hardly long enough to show if there has been any significant change in the mean age of patients or if more older women are now being treated than before. The mean age of 262 ever-married patients who first attended during the first half of the survey (1944–53 city ; 1950–56 county) was 52·5 years while the mean age of the 269 who first attended during the second half (1954–63 city ; 1957–63 county) was 53·9 years. If those attending during the first half are related to the 1951 census and those attending during the second half to the 1961 census, the rate of cervical cancer is 3·5 per 10,000 in both groups. Rates in elderly women (aged 65 and over) are the same in the second half as in the first half (3·3 per 10,000). The rate in young women (aged 25–34) is lower in the second half (0·4 per 10,000) than in the first half (1·4).

Although the Aberdeen survey goes back to 1944 some of the features of the post-war world, such as earlier marriage, more multiple marriages and, if it is so, the tendency to greater sexual freedom in women, all of which might be expected from the evidence of numerous studies to increase rather than decrease the rate of cervical cancer in younger women, have not yet had time to exert an influence. Given a latent period generally estimated to average 30 years, the disease in many of our 571 patients developed in the conditions prevailing in the thirties, twenties or even earlier. Three-quarters of the patients were married before 1935 and nearly half before 1925. The fall in the number of pregnancies since those days, better nutrition and better standards of hygiene may have contributed to lower the rates of cervical cancer in younger women.

*Marital state*

Mortality statistics have shown for many causes of death higher death rates in widowed and single people than in married people of the same age. In the case of cancer of the female genital organs marital state influences the site of the cancer. The unmarried woman is more liable to cancer of the body of the uterus and ovary than either the married or widowed, while the married woman, and particularly the widow, is more liable to die from cancer of the cervix than the unmarried woman, as the Registrar-General (1964) has shown (Table II).

TABLE II.—*Malignant Neoplasms of the Genital Organs: Standardised Mortality Ratios*, 1959, *England and Wales*

(All causes at ages 15 and over = 100)

| Cause of death | Single | Married | Widowed |
|---|---|---|---|
| Cancer of: | | | |
| Cervix uteri | 39 | 101 | 127 |
| Corpus uteri | 139 | 89 | 99 |
| Ovary and Fallopian tubes | 140 | 92 | 96 |

In Aberdeenshire the mean annual rate of cervical cancer is 1·0 per 10,000 single women, 3·3 in the married (or separated) and 4·6 in the widowed (or divorced).

TABLE III.—*Mean Annual Rate of Cervical Cancer per* 10,000 *Women by Marital State: Aberdeen and County*

| | Rate per 10,000 women | | |
|---|---|---|---|
| Age group | Single | Married and separated | Widowed and divorced |
| 25–44 | 0·7 | 1·9 | 5·8 |
| 45–54 | 1·2 | 4·7 | 6·1 |
| 55–64 | 1·4 | 5·6 | 7·0 |
| 65+ | 1·1 | 4·2 | 3·1 |
| Total | 1·0 | 3·3 | 4·6 |

The high rate in the *widowed and divorced* under age 45 may not be entirely an effect of small numbers (Table III). The Registrar-General has also shown that although death rates from cervical cancer are higher in widows (including divorced) than in married women in every age group, the rate is especially high in younger widows (under 45), amounting to twice that for married women of the same age.

The high rate seen in Table III in the widowed compared with the married in the age-group 25–44 could be due to differences within the age group, most widows being at the higher end while most married women are found in the age-group 35–39 which has a lower incidence of cervical cancer. The Aberdeenshire numbers are too small, however, for further age breakdown. The higher rate in the widowed and divorced could also be an effect of excluding those who remarry from the widowed category. Selection for remarriage could favour women who are less likely to develop cancer of the cervix, being perhaps younger, with smaller families or in better health. Differences in occupational status might also affect rates in younger married and widowed women. The higher mortality rates in men aged 25–44 employed in manual rather than non-manual work, and particularly in unskilled work, mean that their widows are predominantly in those socio-economic groups most liable to cervical cancer.

The mean annual rate of squamous cell cancer of the cervix in *single women* in Aberdeenshire is 1·0 per 10,000 aged 25 or over. Their actual number in the present survey is 40, 20 in Aberdeen and 20 in the smaller population of the county, which therefore has the higher rate. This is contrary to the usual finding of a lower incidence of cervical cancer in country areas and may be associated with the higher illegitimacy rates of country compared with urban areas. Two-thirds of the single patients were known to have had one or more pregnancy and of the 10 who said they had no pregnancy two were noted after examination to be " almost certainly not nulliparous ".

| | Number of pregnancies of single women |
|---|---|
| None . . . . | 10 |
| One or two . . . | 21 |
| Three or four . . . | 3 |
| Five or more . . . | 3 |
| Not stated . . . | 3 |
| | 40 |

Thompson (1956), in a study of illegitimate maternities in Aberdeen, has shown that illegitimacy tends to be associated with unskilled, unattractive or menial occupations, and that this may arise from personal or environmental limitations, especially during childhood. Only a rough comparison is possible between the occupations of the single women patients at the time they attended hospital and of the general female population of Aberdeenshire. The Registrar-General (1952) has shown that in 1951 half the gainfully occupied women of all ages in Aberdeenshire were in non-manual work, including clerical, professional and technical work and work in commercial, finance and insurance occupations. This group only provides 15 per cent of patients. Twenty-eight per cent of the population, and 50 per cent of patients, were in personal service work, including domestic work and work in hotels and institutions. Domestic work is the main occupational opening for the countrywoman in Aberdeenshire. The high proportion of patients so employed may be due to the convenience of this type of work to the single woman with a dependent child ; also women who are in fact co-habiting sometimes describe themselves as " housekeepers ". Another 21 per cent of the population and about the same proportion of single patients, did skilled or unskilled manual work. In 5 of the 40 cases the patients' occupations were not known.

The mean age of the single patients was 53·8 years, compared with 53·2 years for ever-married patients. The youngest were 3 parous women who attended with cancer of the cervix in Stages II and III, one at age 30, 3 years after the birth of a child, and 2 at age 32, 14 and 11 years respectively after the birth of a child.

*Occupational class*

The frequency of cancer of the cervix is generally found to vary with socio-economic status, whether this is measured by husband's occupation, income, rent paid, residential area or, in the U.S.A., merely by division into " private " and " clinic " patients. The gradient, rising as socio-economic status falls, appears clearly in the Registrar-General's (1958) standardised mortality ratios for five social classes (Table IV). The classes are broad, however, and contain many occupational groups. When the last three are divided functionally into subclasses, S.M.R's show considerable variation within the three broad categories. In the third section of Table IV occupations are rearranged into 12 slightly more sharply defined socio-economic groups. Their approximate relationship to the 5 social classes is indicated.

TABLE IV.—*Standardised Mortality Ratios* for Cervical Cancer in Married Women aged* 20–64 *by Social Class, Social Sub-class and Socio-economic Group : England and Wales,* 1950–53

| Social class | S.M.R. | Social sub-class | S.M.R. | Socio-economic group | S.M.R. |
|---|---|---|---|---|---|
| I Prof. and managerial | 64 | . | 64 | . Higher admin., prof. and managerial | 65 |
| II Intermediate | 75 | . | 75 | . Other admin., prof. and managerial | 71 |
| | | | | . Farmers | 73 |
| | | | | . Shopkeepers, proprietors and managers of whole-sale businesses | 84 |
| III Skilled workers | 98 | . Clerical workers | 80 | . Shop assistants | 63 |
| | | Other skilled workers | 94 | . Clerical workers | 79 |
| | | Mine-workers | 124 | . Foremen | 89 |
| | | Transport workers | 128 | . Skilled workers | 105 |
| | | Armed Forces (other ranks) | 168* | . Personal service | 106 |
| IV Partly skilled workers | 105 | . Agricultural workers | 81 | . Agricultural workers | 76 |
| | | Others in partly skilled occupations | 111 | . Semi-skilled workers | 112 |
| V Unskilled workers | 134 | . Building and dock labourers | 122 | . Unskilled workers | 135 |
| | | Others in unskilled occupations | 138 | . | |

* A *Standardised Mortality Ratio* (S.M.R.) shows the number of deaths registered in the year of experience as a percentage of those which would have been expected in that year had the sex/age mortality of a standard period operated on the sex/age population of the year of experience.

Table IV shows ratios to be highest in women whose husbands are semi-skilled or unskilled manual workers (but not agricultural workers) or who are in the Armed Forces. The sub-class " transport workers " is broad and its ratio, falling between those for skilled and unskilled manual workers, may reflect this. Shop assistants share with the higher administrative, professional and managerial groups the lowest ratios of all.

The special census tabulations made for the 1951 Aberdeenshire population now make it possible to supplement these mortality ratios with incidence rates. The occupational classification of the present survey is based on the recently expanded and revised version of the socio-economic groups introduced in 1951. Categories were reduced from 15 to 8 to increase numbers for working out rates; As the husband's occupation is seldom stated for widowed and divorced women it was not possible to include them in the analysis by socio-economic group, and Table V shows rates in married women aged 25 and over.

The incidence rates in Table V show much the same occupational gradient as the standardised mortality ratios in Table IV, despite small numbers. Agricultural workers in Aberdeen county, however, have a higher rate than their S.M.R.

TABLE V.—*Mean Annual Rate of Cervical Cancer per 10,000 Married Women, by Socio-economic Group : Aberdeen and County*

| Socio-economic group | Rate per 10,000 | Population | Patients |
|---|---|---|---|
| Employers and managers, professionals | 1·57 | 8,326 | 24 |
| Farmers | 1·78 | 5,081 | 13 |
| Intermediate non-manual workers* | 2·21 | 7,233 | 30 |
| Skilled manual workers and foremen | 3·10 | 22,956 | 127 |
| Semi-skilled and personal service workers | 4·43 | 9,524 | 77 |
| Agricultural workers | 4·48 | 3,345 | 23 |
| Unskilled manual workers | 6·12 | 6,602 | 77 |
| Armed Forces, other ranks | 12·12 | 304 | 7 |
| Not stated | — | 445 | 16 |
| Total | 3·47 | 63,816 | 394 |

* Shop assistants, clerical workers, insurance agents, policemen, draughtsmen and similar "intermediate" occupations.

ranking would indicate, coming between that for semi-skilled and unskilled manual workers. Wives of men in the Armed Forces again have the highest ratio of all, albeit for very small numbers.

Differences in age at marriage and parity could account for some of the differences between the groups. This can be studied in the 1961 10 per cent sample of married women in Aberdeenshire. Assuming a higher risk of cervical cancer in those who marry under age 20 or who have had 5 or more children, Table VI shows how the socio-economic groups are ranked according to this risk. Farmers' and agricultural workers' wives who appear in the city census are included with those in the county, and wives of men in the Armed Forces, being very few in number, are included with the semi-skilled.

TABLE VI.—*Percentage of Married Women in Each Socio-economic Group Who Had 5 or More Children, and Percentage Who Had Married Under Age 20*

| | 1961 population (10% sample) | | % of each soc.-econ. group: With 5 or more children | | % of each soc.-econ. group: Married under age 20 | |
|---|---|---|---|---|---|---|
| Socio-economic group | City | County | City | County | City | County |
| Professional | 133 | 86 | 1·5 | 3·5 | 3·0 | 3·5 |
| Employers and managers | 451 | 303 | 3·8 | 6·6 | 8·0 | 9·2 |
| Intermediate non-manual | 618 | 269 | 3·6 | 5·6 | 7·1 | 9·7 |
| Skilled manual workers and foremen | 1,407 | 692 | 7·4 | 9·0 | 15·2 | 17·0 |
| Semi-skilled and other ranks, Forces | 573 | 318 | 14·1 | 17·6 | 17·6 | 18·6 |
| Unskilled manual workers | 374 | 136 | 16·8 | 22·8 | 20·3 | 23·5 |
| Farmers | 17 | 525 | 14·6 | | 8·7 | |
| Agricultural workers | 20 | 354 | 16·8 | | 25·1 | |

A very similar proportion of wives of farmers and of agricultural workers have 5 or more children. Nearly three times as many wives of agricultural workers as of farmers, however, marry under age 20. In theory, their risk should be greatest and the rate for farmers' wives should be higher than it appears to be from Table V, were it not for the unexplained protective influences of living in a rural area. It is interesting to note that the lower rate of cervical cancer in married women in

Aberdeen county is achieved in spite of the greater frequency of early marriage and large families in the county than in the city in all the socio-economic groups.

### *Age at Marriage and Number of Live-born Children*

That early marriage is more common among patients with cervical cancer than among controls has been shown by many studies. Wynder *et al.* (1954) thought that early marriage, early coitus and re-marriage all might increase exposure to males with poor penile hygiene. Lombard and Potter (1950) suggested a greater susceptibility of immature tissue to excessive hormonal stimulation. Rotkin (1962) thought that a single application of some substance or particle donated by the male in one coital act, and to which the female is most susceptible in adolescence, appears sufficient for carcinogenic contamination. Dorn (1959) points out that it is not consistently true that the *incidence rate* of cervical cancer is higher for populations in which a high percentage of women marry under age 20. He cites Sephardic-Oriental and Ashkenazic Jews who have a similar incidence although the percentage marrying under 20 is more than twice as great in the former as in the latter. Rates are similar for New York and Israeli Jews although the proportion marrying under age 20 differs widely. New York Negroes have a very much higher rate than Sephardic-Oriental Jews, although a higher proportion of the latter marry under 20. These inconsistencies and the variation in the estimated relative risk of cancer of the cervix in the various population groups may well be due to the defects of retrospective studies to which Dorn refers, for example, that the persons included are usually selected by an unknown method of sampling from an unspecified population. It may also be that consistent results cannot be expected from populations of such diverse social, genetic and environmental backgrounds as Negroes in New York and Jews from the Mediterranean areas. For instance, early marriage may be less of a hazard in populations with a good standard of penile hygiene such as that achieved by universal male circumcision. Such factors as age at marriage are only indicators which may be differently related to the " cause " in different cultures.

The two related variables, age at marriage and number of pregnancies, have been studied conjointly by several authors. Wynder *et al.* (1954) cross-tabulated for age at marriage and attempted to control for social class by interviewing a predominantly " clinic " population. He found no difference in the number of pregnancies of women with cervical cancer and a comparable group with other gynaecological complaints when these two major variables were controlled in this way. Boyd and Doll (1964) compared patients with controls of about the same age chosen from the same hospitals, some controls being patients with gynaecological complaints and another group with medical or surgical conditions. They found that after allowing for possible parity effects the difference between the groups in respect of age at marriage remained significant. Comparison of the number of children after standardisation for age at marriage, however, suggested that the differences noted with respect to parity were a result of the differences in age at marriage and provided no evidence of an independent effect of parity.

In a study made for the British Empire Cancer Campaign Stocks (1957) used women in age-groups from 45–74 without cancer seen in a Liverpool hospital as controls for women who had died from certain cancers in Liverpool and adjoining parts of Lancashire. For cancer of the cervix, he found at each age an excess of women who had married before age 20, and among women under age 55 the number with 5 or more confinements exceeded the expected number for those who married before 25. To check his findings he studied the distribution by age at marriage and fertility conjointly of all women who died of cancer of the cervix not only around Liverpool and also in North Wales and Cheshire, whether or not they had been in hospital, and compared them with women from the same areas dying from stomach cancer. He found that in North Wales and Cheshire there was no relation with family size after standardisation for age at marriage. In the Liverpool area, however, there still persisted an association between the incidence of the disease and high fertility as seen in his first tabulation.

Lundin *et al.* (1964) studied the socio-economic distribution of cervical cancer in relation to early marriage and pregnancy by comparing cases found in a cytological screening survey in Memphis, Tennessee, and a random 2·8 per cent sample of women who had taken part in the survey but in whom there was no evidence of uterine cancer. They showed a consistent and strong association between cancer of the cervix and early marriage for white (though not for non-white) women. However, when adjustment was made for age and pregnancy history in the whites, expected and observed numbers in the different age at marriage categories were almost equal. They concluded that in married white women pregnancy, and especially early pregnancy, is sufficient to account for the observed association between early marriage and cancer of the cervix.

It is now possible for the first time in this country to compare the age at marriage distribution of patients with cervical cancer with that of the general population of the area, standardised to the composition of the patients in regard to age, family size and husband's socio-economic group simultaneously. The control population was derived from the 1961 10 per cent sample. To check the results, a further tabulation was made in which patients *under age* 50, who first attended hospital between 1944–53 were compared with the 1951 census population, while patients of *all* ages first attending between 1954–63 were compared with the 1961 sample. (The difference in the age of the two groups results from the need to match the available census data.) The expected numbers from these two populations were combined and compared with the combined observed number of patients. This showed the same pattern as the first tabulation, suggesting that the population has not changed sufficiently between 1951 and 1961 to affect results. The tabulations refer to once-married currently married patients and to live-born children to match the census population. It is unfortunate that widowed and divorced patients and those married more than once have had to be excluded, as these comprise nearly a third of the ever-married patients. Despite this limitation, the use of census material and the close standardisation of the population to the patients make the Aberdeenshire data of unique interest (Table VII).

Women marry earlier today than in the 1920's and 1930's when many of the patients in the present survey were marrying. To get a correct picture, cancer patients must be compared with women of the same age ; this is done in columns (a) and (b). These show that considerably more patients than expected had married under age 20, while fewer than expected had married at 25 or over, the pattern generally found when patients and controls of the same age are compared. This result could be affected by social class differences between patients and population, women in the lower socio-economic groups marrying relatively early and being more liable to cervical cancer than women in the higher socio-economic

TABLE VII.—*Age at Marriage : Observed and Expected Numbers*

| Age at marriage | No. of cancer patients observed (a) | Expected number of patients when standardised for : Present age only (b) | Age and socio-economic group (c) | Age and number of children (d) | Age, number of children and soc.-econ. group (e) |
|---|---|---|---|---|---|
| <20 | 78 | 39 | 48 | 63 | 72 |
| 20– | 135 | 140 | 145 | 156 | 161 |
| 25– | 81 | 94 | 83 | 79 | 65 |
| 30– | 18 | 34 | 31 | 22 | 22 |
| 35+ | 17 | 22 | 22 | 9 | 9 |
| Total : | 329 | 329 | 329 | 329 | 329 |

groups. Column (c) therefore shows the expected age at marriage distribution of the population when it is of the same age and socio-economic status as the patients. This reduces the gap between the observed and expected numbers marrying under 20 and at the combined ages 25 and over, while slightly increasing the discrepancy at 20–24. When the number of live-born children is substituted for socio-economic group in column (d), these trends are repeated, the gap between observed and expected marrying under 20 being further reduced. Finally, in column (e) all three control factors are combined to provide a joint standardisation. The excess of patients marrying under age 20 compared to the population is almost eliminated, while the deficiency of patients marrying at 20–24 is increased. At age 25 and over more cases are observed (116) than are expected (96), the reverse of what is usually found. The differences between columns (a) and (e) are significant, although not highly so ($p < 0{\cdot}05$).

These unusual results are difficult to interpret. They might be questioned on the grounds of too elaborate a standardisation procedure for the small numbers involved. It is relevant to note here, however, that a comparison in the second part of this study (Aitken-Swan and Baird, 1966) of age at marriage of patients with pre-clinical cancer of the cervix and controls matched for age and parity shows marriage under age 20 to be equally frequent in both groups.

The variable which had the most effect in reducing the difference between the observed and expected number of cases was the number of children the woman had borne. Indeed, there is some suggestion that what was remarkable about the cancer patients was not their age at marriage but the large number of children their years of marriage had produced. In Table VIII the same standardising procedure is followed to discover whether, when allowance is made for other factors, the excess of women of high parity is eliminated.

Table VIII, columns (a) and (b), shows that when women of the same age are compared fewer childless patients and fewer with 1, 2 or 3 children than expected are seen. With 4 or more children a sudden reversal appears, and in fact in cancer patients families of 7 or more children are 5 times as numerous as expected. The addition of socio-economic group to age as a standardising factor in column (c) does not alter the expected numbers appreciably. Column (d) shows the expected numbers when women of the same age and age at marriage are compared. In most categories the difference between observed and expected numbers becomes less although the same pattern is seen, a marked deficit of patients with up to 3 children and an excess with 4 or more. Finally, contrary to the findings of most

TABLE VIII.—*Number of Live-born Children : Observed and Expected*

| Number of live-born children | No. of cancer patients observed (a) | Expected number of patients when standardised for : Present age only (b) | Age and socio-economic group (c) | Age and age at marriage (d) | Age, age at marriage and soc.-econ. group (e) |
|---|---|---|---|---|---|
| 0 | 17 | 48 | 46 | 41 | 33 |
| 1 | 38 | 68 | 64 | 60 | 57 |
| 2 | 71 | 87 | 83 | 83 | 84 |
| 3 | 48 | 58 | 59 | 61 | 60 |
| 4 | 51 | 30 | 32 | 34 | 37 |
| 5 | 27 | 17 | 18 | 21 | 24 |
| 6 | 19 | 10 | 13 | 13 | 16 |
| 7+ | 58 | 11 | 14 | 16 | 18 |
| Total : | 329 | 329 | 329 | 329 | 329 |

other studies, column (e) shows that a highly significant difference between the observed and expected number of children still remains after standardisation for present age, age at marriage and husband's socio-economic group simultaneously. The observed number of childless patients is about half that expected, while the number with 7 or more children is over 3 times as high in the cancer patients as in the standardised population.

Large families were more common in the first third of this century than they are today (44 of the 58 patients with 7 or more children were married before 1930), but they still help to distinguish a group with particularly unfavourable social circumstances and a way of life likely to predispose to cancer of the cervix, with little opportunity for hygiene and other protective influences. This could account for the great excess of women with 7 or more children among patients with cervical cancer compared with the expected number. The mean age of the 58 patients with 7 or more children is higher—55 years—than that for all once-married, currently married patients (51 years), and 16 of the 58 were aged under 50. These younger women were concentrated in the semi-skilled and unskilled manual classes while all 58 are more evenly spread among the various categories of manual workers. As the survey covers 20 years, it is worth noting that the patients with 7 or more children are not to be found mainly in the earlier years ; in fact, 28 attended in the first ten years of the survey compared with 30 in the second ten years.

The risk of getting cancer of the cervix has been shown to rise as socio-economic status falls (Table V). Wives of non-manual workers, who have the lowest risk, marry later and have fewer children than wives of manual workers (Table VI). Fig. 2 seeks to show what happens to the incidence rate when each group behaves uncharacteristically in this respect, that is, when the wives of non-manual workers marry early and have large families and when the wives of manual workers marry later in life and have few children. As numbers are not large enough to show this for each socio-economic group separately, they are grouped into (1) a low risk group, comprising wives of professional men, managers and farmers, and intermediate non-manual workers, and (2) a high risk group, comprising wives of skilled, semi-skilled and unskilled manual workers, agricultural workers and Armed Forces.

The top half of Fig. 2 shows age at marriage when standardised for number of live-born children. The lower half shows the number of live-born children when

FIG. 2.—Incidence rates of cancer of the cervix by (a) age at marriage and (b) number of live-born children, in low and higher risk groups.

similarly standardised for age at marriage. The difference in the rates for the two groups is by definition expected. The top diagram shows that women in the high risk group are at a disadvantage in comparison with women in the low risk group regardless of age at marriage ; for instance, the high risk group marrying over the age of 20 have the same rate as the low risk group marrying under age 20. This is doubtless associated with the fact that as social class declines age at marriage is a less reliable indicator of age at first coitus, as will be seen in the following paper (Aitken-Swan and Baird, 1966) of this article. Similarly, as the bottom diagram shows, women in the high risk group with 3 or 4 children have a higher rate than women in the low risk group with 5 or more. There is no difference in favour of the high risk group except when those with 0–2 children are compared with the low risk group with 5 or more. The diagram indicates again the many aspects of social class differences in cervical cancer.

### DISCUSSION

The social and environmental factors which appear to play a predisposing part in the aetiology of cervical cancer are not uncommon. Nevertheless, cancer of the cervix is not a common disease, even among those who marry young, who have numerous children, several partners, low standards of hygiene or who belong to the lower socio-economic groups. The number of women attending hospital for the first time with cancer of the cervix is relatively small in any one year ; in Aberdeenshire, they average 4 in 10,000 ever-married women aged 25 and over, or 1 in 10,000 single women. Our type of data cannot show accurately the probability of any one woman developing the disease during her lifetime but, if certain assumptions are made, this chance in ever-married women might be about 1 in 50, at a rough estimate from the data in Table I. This is similar to a finding reported from New York State (Randall *et al.*, 1950), which showed the lifetime probability of developing cancer of the cervix to be 2·2 per 100 women. We do not know all the factors that influence the selection of this one from the 49 others who do not get the disease, although similar in many respects, doing much the same things and exposed to the same conditions of life. Only some of the complex combination of factors can be identified by epidemiological and social study. In this article we have tried to show some of the ways in which patients with cervical cancer differ from the general population of Aberdeenshire and how the incidence of the disease varies in different population groups, both here and elsewhere.

The amount of cancer of the cervix is lower in Aberdeenshire than in Denmark, with which it is compared in Fig. 1. Social and environmental differences and differences in sexual *mores* rather than the efficiency of Danish cancer registration are likely to account for this. The lower incidence rates from ages 50–64 in the Liverpool and Birmingham regions compared with Aberdeenshire are surprising. Greater urbanisation promotes the conditions conducive to cancer of the cervix, and a highly industrial area which includes a great sea-port through which many transients are passing, might be expected from Stocks' data to have a higher rate of cervical cancer than a predominantly rural county such as Aberdeenshire, even including Aberdeen. The stressful conditions assumed to promote cancer of the cervix have not produced more of the disease in Liverpool than in Aberdeenshire—the peak rate in Liverpool is lower than in Aberdeenshire—but they have produced it earlier in life, in the fifth rather than the sixth decade. In Denmark as in Liverpool peak rates are earlier in life than in Aberdeenshire but Denmark has a much higher incidence of the disease as well.

A fall is generally, though not invariably, found in the incidence of cervical cancer after middle age. Rates in the Birmingham region are unusual in that they show a fluctuating rise to age 70.

In some patients the interval from marriage to the diagnosis of cancer is so great as to suggest that in elderly women the disease may have a different aetiology. For example, among Aberdeenshire patients known to have married or to have had a pregnancy at age 16 or 17 (so that the margin of error regarding age at " first stimulus " is small), the interval to the diagnosis of clinical cancer was 13 years in one case and 56 years in another. The former case could be an example of the most common type of cervical cancer, that associated with coitus and possibly promoted by a virus or other carcinogenic agent and characteristically showing itself some 30 years after " first stimulus " (i.e. in middle age). Elderly patients, on the other hand, might have escaped cancer at the age when the type associated with coitus is most common, but as they become older the tendency to develop cancer in a variety of sites increases. There is no reason why this degenerative type of cancer should not arise in the cervix. The precise site of origin and extent of the lesion may be more difficult to define because of anatomical changes, such as shrinkage of the cervix and the vaginal vault. The evidence suggests, however, that the aetiology of cancer of the cervix in the elderly differs considerably from that in younger women. It may be that in those areas which show increasing incidence rates with age instead of the customary peak in the middle years, some factor is reducing the amount of cancer of the type associated with coitus while the rate of degenerative cancers in the elderly remains the same.

We have shown that the well-known social class gradient in death rates from cancer of the cervix also applies to the incidence of the disease. For the first time incidence rates are shown in 8 socio-economic groups (Table V). Two findings of particular interest are the difference in rates between wives of farmers and agricultural workers, and the particularly high rate in wives of men in the Armed Forces. The category " farmers " includes small crofters and others whose rate might be expected to be nearer to that of agricultural workers than of the non-manual groups. Rates so divergent as 1·8 (farmers) and 4·5 (agricultural workers) suggest important differences to be found in their ways of life. The high rate in wives of men in the Armed Forces must be accepted with caution because of small numbers, but it is interesting that in Table IV these wives have the highest standardised mortality ratio for cancer of the cervix of any of the social sub-classes listed, although again this ratio is based on small numbers (32 deaths). However, the possibility that cancer of the cervix may be higher in wives of Servicemen than in other groups suggests that it might be profitable to look at the incidence rate in wives of men away from home as a group, that is, in addition to Servicemen, seamen, those working away for long periods, long-distance drivers and others who might be at greater risk of contracting the carcinogenic agent.

There is a striking difference between the findings of this study and other studies on age at marriage and family size of patients with cervical cancer compared with non-patients. Previous studies have shown that more patients than controls of the same age (and perhaps social class) had married under age 20, and that although more patients than controls have large families, this is an effect of their earlier marriage and that the difference disappears when women married at the same age are compared.

In this study, the number of cancer patients who married before the age of 20 was only slightly higher than the number expected in a population of the same age, social class composition and the same family size as the patients. Fewer patients than expected had small families (3 children or less) and more than expected had 4 or more. Families containing 7 or more children were actually three times as common in the cancer group as expected. Our results require confirmation since we may have taken the process of standardisation too far for the relatively small numbers involved. Also, widowed, divorced women and those married more than once have been excluded and their reproductive pattern (age at marriage, family size, etc.) may be different from that of the once-married. At the same time, the method of comparing patients with the population from which they come has advantages over the selection of any other control group. Control groups are difficult to select. For example, bias could result from the use of " hospital " controls and particularly of patients in gynaecological wards as controls for women with cervical cancer. Many illnesses have a social class bias. Gynaecological patients tend to be of higher parity than other women and, as we have seen, parity is closely related to age at marriage and social class. Nor are the many facets of social class adequately controlled by choosing controls from among patients attending the same hospital or clinic as the cancer patients, even if the hospital or clinic predominantly serves a particular section of the population. More exact matching is necessary if differences between patients and controls are to stand out that are not due in fact to comparing individuals with dissimilar standards of living and ways of life.

This part of the study has shown the incidence of cancer of the cervix in Aberdeenshire and how it is distributed in the city and country areas and in the various sub-groups of the population. The following paper will describe some of the characteristics of patients with clinical cancer in more detail, and more particularly presents the results of a detailed analysis of patients with pre-clinical cancer found in the course of cytological screening. Finally, the aetiological implications of the findings will be discussed.

### SUMMARY

Aberdeenshire patients with cancer of the cervix are related to the census population for the area to produce mean annual incidence rates for groups of women of different age, marital and socio-economic status. In any year, an average of 4 in 10,000 ever-married women aged 25 and over, or 1 in 10,000 single women can be expected to attend hospital for the first time with the disease. Rates are higher in the city than in the county of Aberdeen, in widowed and divorced women and in the lower socio-economic groups.

For the first time it is possible to compare age at marriage and family size in married patients and in the census population when standardised for two additional factors, namely, present age of patient and husband's socio-economic group (4 variables simultaneously). Contrary to the usual findings, there is no significant excess of patients marrying under age 20. Families of 4 or more children were more frequent in patients with cervical cancer than in the general population ; very large families (7 children or more) were three times as common.

### REFERENCES

Aitken-Swan, Jean and Baird, D.—(1966) *Br. J. Cancer*, **20**, 642.
Boyd, J. T. and Doll, R.—(1964) *Br. J. Cancer*, **18**, 419.
Clemmesen, J.—(1965) *Acta path. microbiol. scand.*, Suppl. 174, ii, 245.
Dorn, H. F.—(1959) *New Engl. J. Med.*, **261**, 12, 571.
Lawson, J. G.—(1957) *J. Obstet. Gynaec. Br. Commonw.*, **64**, 4, 488.
Lombard, H. L. and Potter, E. A.—(1950) *Cancer, N.Y.*, **3**, 960.
Lundin, F. E. Jr., Erickson, C. C. and Sprunt, D. H.—(1964) *Publ. Hlth Monogr.* No. 73. U.S. Dept. of Health, Education and Welfare.
Randall, C. L., Gerhardt, P. R., Handy, V. H. and Kraus, A. S.—(1950) *Am. J. Obstet. Gynec.*, **68**, 1378.
Registrar-General.—(1950–64) Annual Reports for Scotland, Edinburgh (H.M. Stationery Office).—(1952) Census of 1951 One Per Cent Sample Tables, Part I, London (H.M. Stationery Office).—(1957) Statistical Review of England and Wales, 1952 : Supplement on Cancer, London (H.M. Stationery Office).—(1958) Decennial Supplement for 1951 : Occupational Mortality, Part II, Vol. 1, Commentary, London (H.M. Stationery Office).—(1964) Statistical Review of England and Wales, 1961, part III, London (H.M. Stationery Office).
Rotkin, I. D.—(1962) *J. Am. med. Ass.*, **179**, 486.
Stocks, P.—(1955) *Br. J. Cancer*, **9**, 487.—(1957) *Rep. Br. Emp. Cancer Campn*, **35**, Supplement to Part II.
Thompson, B.—(1956) *Br. J. prev. soc. Med.*, **10**, 75.
Wilkes, E.—(1964) *Lancet*, i, 1379.
Wynder, E. L., Cornfield, J., Schroff, P. D. and Doraiswami, K. R.—(1954) *Am. J. Obstet. Gynec.*, **68**, 1016.